# A quantitative model for charge carrier transport, trapping and recombination in nanocrystal-based solar cells

**DOI:** 10.1038/ncomms7180

**Published:** 2015-01-27

**Authors:** Deniz Bozyigit, Weyde M. M. Lin, Nuri Yazdani, Olesya Yarema, Vanessa Wood

**Affiliations:** 1 Laboratory for Nanoelectronics, Department of Information Technology and Electrical Engineering, ETH Zurich 8092, Switzerland

## Abstract

Improving devices incorporating solution-processed nanocrystal-based semiconductors requires a better understanding of charge transport in these complex, inorganic–organic materials. Here we perform a systematic study on PbS nanocrystal-based diodes using temperature-dependent current–voltage characterization and thermal admittance spectroscopy to develop a model for charge transport that is applicable to different nanocrystal-solids and device architectures. Our analysis confirms that charge transport occurs in states that derive from the quantum-confined electronic levels of the individual nanocrystals and is governed by diffusion-controlled trap-assisted recombination. The current is limited not by the Schottky effect, but by Fermi-level pinning because of trap states that is independent of the electrode–nanocrystal interface. Our model successfully explains the non-trivial trends in charge transport as a function of nanocrystal size and the origins of the trade-offs facing the optimization of nanocrystal-based solar cells. We use the insights from our charge transport model to formulate design guidelines for engineering higher-performance nanocrystal-based devices.

## Abstract (web summary)

Colloidal nanocrystals could help improve the performance of the next generation of solar cells, but a model that fully describes the electronic behaviour of such devices is missing. Bozyigit *et al*. now develop a quantitative model for charge transport in these solar cells.

## Graphical abstract

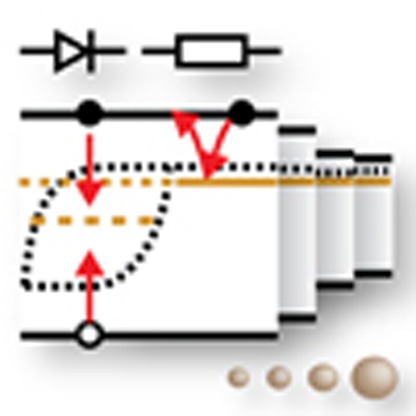

Solar cells incorporating nanocrystals (NCs) hold promise for third-generation solar cells, offering the possibility for low-cost manufacturing of cells that overcome the Shockley-Queisser limit^1^,^2^,^3^ through multi-junction cells^4^ and multiple exciton generation^5^. The constituent NCs and the NC-based absorptive layers can be solution processed using inexpensive, large area compatible techniques and many NCs exhibit high-absorption cross-sections compared with the bulk, such that less active material is required^6^. Furthermore, NCs offer a tuneable bandgap based on material chemistry and size, which presents the possibility for optimal bandgap selection and fabrication of multi-junction cells^7^,^8^.

Despite the excellent optical properties of NCs, the best certified power conversion efficiency of a NC-based solar cell is currently close to just 9% (refs 9,10). The recent efficiency improvements were achieved by surface treatments of the NCs before or during their deposition into solids. Different explanations have been put forward as to why surface treatments improve performance. Many reports propose that surface treatments passivate trap states, which are expected to reduce conductivity and serve as recombination centres^11^,^12^,^13^. Recent reports, which led to the current record performing device, determined that the role of surface treatments is mainly in the alignment of energy levels through surface dipoles on the NCs^10^,^14^. Although the impact of surface treatments on device performance is undisputed, no consistent explanation has been formed as to which physical processes limit the performance and how trap states are involved^15^,^16^,^17^,^18^,^19^.

To rationally assess the impact of different fabrication techniques, it is necessary to develop a consistent and predictive model of charge transport in NC-based solar cells. Any such model must quantitatively explain the dark current, which is one of the fundamental and, conveniently, most experimentally accessible characteristics of a diode. The dark current in a diode can provide direct insight into the charge transport, trapping and recombination processes that play an important role in the power conversion efficiency of a solar cell. In particular, the dark current places an upper bound on the maximum power point of the solar cell and the achievable open-circuit voltage^20^. Understanding the physical processes that determine the dark current in a NC-based solar cell would enable us to assess the origins of performance limitations in these devices and develop guidelines for achieving higher performance.

Early studies of the dark current of NC-based diodes presented temperature-dependent measurements^15^,^21^, but, by changing only temperature, it is not possible to obtain sufficient information to uniquely identify the physical processes that govern charge transport. Data in these studies were therefore explained using variations of the majority carrier emission theory developed for single-crystalline semiconductors that is known as the Schottky diode model. Other studies investigated the dark current as a function of NC size^22^,^23^,^24^. Without varying the temperature, these studies had to rely in their analysis on the assumption that, for a process such as the diode current, described by the form *A* exp[−*E*_A_/*kT*], the prefactor (*A*) and the activation energy (*E*_A_) are not correlated. In fact, correlation between the prefactor and activation energy is very common in disordered and defect-rich semiconductors and is known as the Meyer–Neldel rule^25^.

In the following, we carry out a systematic study investigating the dark current in metal–semiconductor–metal (MSM) diodes made from PbS NCs varying both the bandgap of the NCs and the temperature. Using thermal admittance spectroscopy (TAS) to measure the energy and number density of trap states, we can decouple the influence of the bandgap, trap states and temperature to uniquely identify the physical processes that govern the different regimes of the dark current. We use our experimental data to develop a self-consistent and quantitative model of the diode current.

## Results

### Device fabrication and photovoltaic characterization

We synthesize a series of different sized PbS NCs according to the method by Hines *et al*.^26^ From optical absorption measurements of the NCs in solution (Supplementary Fig. 1a), we determine the optical bandgap (*E*_g_), which we take as the peak of the lowest energy exciton transition. Following ref. 27, we then use the optical bandgap to determine that the radii of the NCs range from *r*=1.17 nm (for *E*_g_=1.85 eV) to *r*=3.0 nm (for *E*_g_=0.79 eV), which is consistent with the transmission electron microscope (TEM) images in Supplementary Fig. 1b.

**Figure 1:**
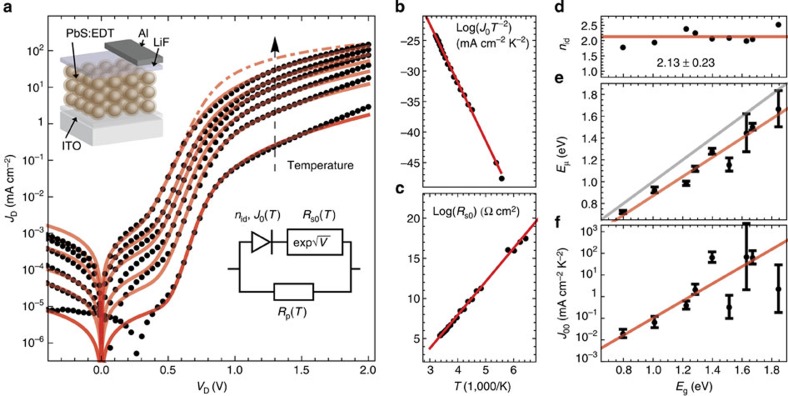
Temperature-dependent current-voltage (IVT) measurements in dependence of NC bandgap (*E*_g_). (**a**) IVT measurement of a metal–semiconductor–metal diode (inset in top left) with *E*_g_=1.69 eV for temperatures between 205 and 295 K (black dots). Fits of the analytical model (red lines) based on the circuit (inset in bottom right) with the saturation current (*J*_0_), series resistance (*R*_s0_) and shunt resistance (*R*_p_) as parameters. For comparison, a fit with a constant series resistance at 300 K is shown (dash–dotted red line). (**b**) Arrhenius-type plot for *J*_0_ (black dots) where a linear fit (red line) gives the mobility bandgap (*E*_μ_) and the reduced saturation current (*J*_00_). (**c**) Arrhenius-type plot for *R*_s0_ (black dots) where a linear fit (red line) gives the activation energy (*E*_rs_) and prefactor (*R*_s00_). (**d**) Ideality factor (*n*_id_) determined at room temperature for *E*_g_ between 0.79 and 1.85 eV. (**e**,**f**) *E*_μ_ and *J*_00_ for different *E*_g_, determined as shown in **b**. As a guide to the eye, the grey line shows *E*_μ_=*E*_g_. Error bars are the standard error from the regressions in **b** and **c**.

We fabricate MSM diodes as depicted in the inset of Fig. 1a and detailed in the Methods section. For each NC size, a NC solid is assembled on an indium tin oxide-coated glass substrate by a layer-by-layer dip-coating procedure using 1,2-ethanedithiol (EDT) in acetonitrile as a crosslinking agent. All PbS NC solids are prepared to a thickness of *d*=100 nm. The diodes are completed with a LiF(1 nm)/Al(100 nm)/Ag(250 nm) top electrode. Results from characterization of each MSM diode under AM1.5 illumination are shown in Supplementary Fig. 2.

To show the generality of our model, we further fabricate and characterize a MSM diode with 1,4-benzenedithiol as a crosslinking agent and a heterojunction device, where the NCs are sandwiched between indium tin oxide/TiO2 and MoOx/Au electrodes. The results of these devices are discussed after developing the model of charge transport based on the analysis of the NC size series in the MSM diode architecture.

### Temperature-dependent current–voltage (IVT) measurements

We perform IVT characterization of each MSM diode in the dark. Figure 1a shows a representative IVT measurements for temperatures between 205 and 295 K for a diode made from NCs with *E*_g_=1.69 eV. As we do not know *a priori* which physical processes determine the diode current, we fit our data with the Shockley diode equation, which is the most general equation for the current through a system with selective transport (that is, a diode). This equation makes no assumptions regarding the underlying charge carrier physics, describing not only semiconductor diodes governed by band transport or variable range hopping, but also more general electrochemical systems^28^. The dependence of the fitting parameters on temperature and bandgap will allow us to determine which physical processes dominate the behaviour of the diode.

Based on the equivalent circuit in Fig. 1a, we write the Shockley diode equation as:


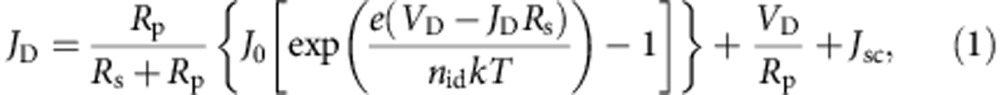


where *J*_D_ and *V*_D_ are the device current and voltage, *R*_s_ the series resistance, *R*_p_ the shunt resistance, and *n*_id_ the ideality factor of the diode. *J*_0_ is the saturation current given by (ref. 20):


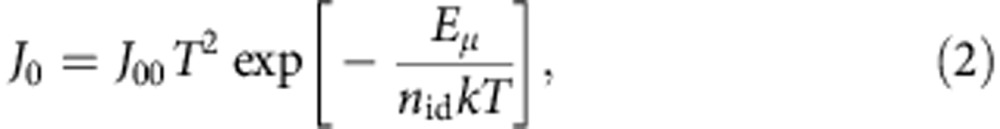


where *E*_μ_ is the mobility bandgap of the free charge carriers and the prefactor *J*_00_ is the reduced saturation current in units of mA cm^−2^ K^−2^. Equation (2) expresses that the diode current is thermally activated, and different physical models ascribe different interpretations to *E*_μ_.

In Fig. 1a, we find that a series resistance causes a roll-off in the large forward bias region (*V*_D_>1 V). We consider a series resistance of the form:


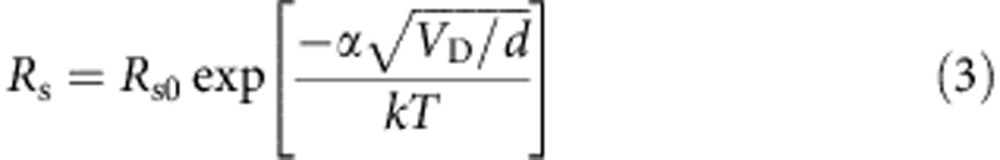


where *d* is the NC film thickness and *α* is a constant, and


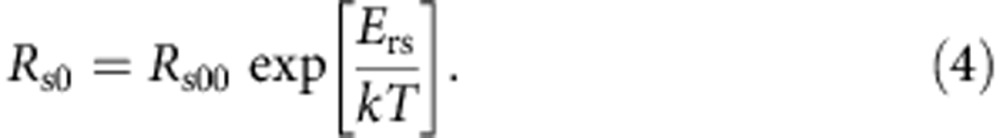


*E*_rs_ is the thermal activation energy of the series resistance and *R*_s00_ a constant prefactor. Equations (3 and 4) describe a process that is based on the electrostatic lowering of a barrier, such as the Schottky effect or the Poole-Frenkel (PF) model^29^. Our selection of the mathematical form of equation (4) for the series resistance is motivated by the excellent fit to the data (Fig. 1a). For comparison, we plot the diode current assuming a constant series resistance (dash–dot red line in Fig. 1a), which does not reproduce the data well for *V*_D_>1 V. Later in this report, we independently confirm the validity of a barrier-lowering model.

In Fig. 1a, we demonstrate the fitting procedure using the data set for NCs with *E*_g_=1.69 eV. First, we extract the ideality factor (*n*_id_=2.06) from the room temperature measurement, then we fit the remaining parameters *J*_0_, *R*_s0_, and *R*_p_ at each temperature. From the Arrhenius plot of *J*_0_ (Fig. 1b), we extract *E*_μ_=1.50 eV and *J*_00_=65.3 mA cm^−2^ K^−2^. Similarly, we use an Arrhenius plot of *R*_s0_ (Fig. 1c) to extract *E*_rs_=0.35 eV and *R*_s00_=2.1 Ω cm^2^. If not otherwise noted, we use *α*=1.96 × 10^−4^ eV V^−1/2^ cm^1/2^ for all fits, the physical significance of which is discussed below. The excellent quality of the fit (Fig. 1a–c) justifies the mathematical description (equations (1, 2, 3, 4)). No reduction in the number of fitting parameters is possible without significantly degrading the quality of fit. A more detailed evaluation of the goodness of fit is given in Supplementary Note 1.

**Figure 2:**
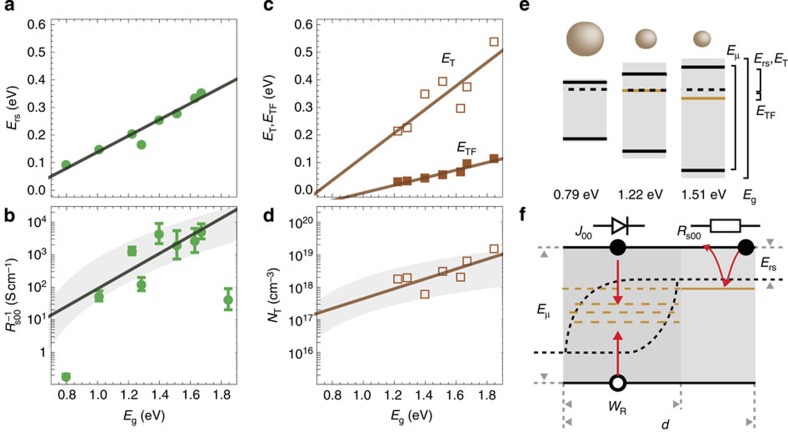
Analysis of series resistance and trap characteristics. (**a**) Activation energy (*E*_rs_) and (**b**) prefactor (*R*_s00_) of the series resistance as a function of NC optical bandgap (*E*_g_) determined by temperature-dependent current–voltage measurements as shown in Fig. 1. Linear fits appear as a solid black line. Shaded grey region in **b** gives *R*^−1^_s00_ predicted by equation (11) using calculated mobilities (Fig. 3e). Error bars are the standard error from the regressions in Fig. 1c. (**c**) The trap-state activation energy (*E*_T_) and the energy difference between Fermi-level and trap energy (*E*_TF_) determined by thermal admittance spectroscopy (TAS) follow a linear trend (solid lines) with the optical bandgap (*E*_g_). (**d**) Trap-state density (*N*_T_) determined by TAS increases with smaller NCs (larger *E*_g_). For reference, a density of 0.1–0.01 traps per NC is plotted in grey. (**e**) Schematic energy diagrams for NC solids composed of different sized NCs showing the optical bandgap (grey), the mobility bandgap (black solid), the position of Fermi-level (dashed) and the position of trap energy (orange). (**f**) Schematic of charge carrier transport in PbS NC-based MSM diodes in forward bias. In the region (left), where the quasi-Fermi-levels for electrons and holes (dashed) are separated, electrons and holes recombine via trap states controlling the diode current in the dark. In the region (right), where the Fermi-level is pinned by trap-states, the current is limited by Poole-Frenkel type conduction at high biases (>1 V).

We perform the same measurements and analysis for devices with each of the different NC sizes. We find that: the ideality factor is close to 2 (*n*_id_=2.13±0.23) for all bandgaps (Fig. 1d); the mobility bandgap follows the optical bandgap (*E*_μ_=0.88 *E*_g_; Fig. 1e); the reduced saturation current increases exponentially with bandgap (log(*J*_00_)=9.15 *E*_g_−11.4; Fig. 1f); the thermal activation energy of the series resistance (*E*_rs_) increases linearly (*E*_rs_=0.29 *E*_g_−0.16; Fig. 2a); and the prefactor of the series resistance (*R*_s00_) decreases exponentially with bandgap (log(*R*^−1^_s00_)=6.23 *E*_g_−1.75; Fig. 2b). A summary of all fits is given in Table 1.

### Characterization of trap-state energies and densities

As trap states can play an essential role in charge transport by changing the effective mobility and serving as recombination centres, we characterize the trap-state density for devices with *E*_g_≥1.2 eV by TAS measurements^30^ as described in ref. 18. We find a discrete trap state with a measured trap-state activation energy (*E*_T_) that increases linearly with bandgap (*E*_T_=0.43 *E*_g_−0.31; Fig. 2c) and a density of trap states (*N*_T_) that increases with bandgap (log(*N*_T_)=3.49 *E*_g_+37.1; Fig. 2d)^31^.

We observe a capacitive response that is indicative of a large trap-state density that pins the Fermi-level close to the trap energy^31^. The model of a non-resonant TAS response allows us to also extract the distance between the trap level and Fermi-level (*E*_FT_=0.14 *E*_g_−0.15; Fig. 2c). In the values for *N*_T_ plotted in Fig. 2d, we have accounted for the pinning of the Fermi-level, as the standard TAS method, which assumes (*E*_FT_=0), would lead to a large underestimation of the trap-state density when *E*_FT_>*kT* (ref. 31).

The implications of the number density of traps and their energetic position for the dark current and solar cell performance are discussed in the following sections. Although understanding the origin of the trap states is not the aim of this work, here we briefly put the observed trends into the context of what is known about trap states in PbS. Comparable discrete, deep trap states have been previously observed experimentally in PbS NC solids that are crosslinked using EDT in field effect transistors (FETs)^19^, solar cells^18^,^32^, photodetectors^17^ and by scanning tunnelling spectroscopy^33^. We find that the trap-state density increases significantly for decreasing NC size. As we are not aware of an established theoretical framework to explain this trend, we show in Fig. 2d that the measured trap-state density lies in the range of 0.1–0.01 traps per NC (grey area). In terms of the energetic position of the traps, we find that increasing the bandgap of the NCs from 1.22 to 1.85 eV increases the energy depth of the traps from 0.2 to 0.5 eV. Theoretical calculations have shown that a stoichiometry with slight excess of lead favours a donor-type defect at around 0.4 eV below the conduction band^34^. Although ref. 34 does not calculate the size dependence of the trap-state energy, ref. 33 shows an increase of the trap energy with larger bandgap (smaller size), similar to our findings.

### The charge transport model

We now use the parameters and their bandgap dependence determined from the IVT and TAS characterization to develop a physical model for charge transport in the NC diode. In this section, we investigate which physical processes govern the diode current in the small forward bias regime, where the diode current increases exponentially, and, in the following section, we discuss the origins of the series resistance that limits the diode current in the large forward bias regime.

There are three common physical models for the current in a semiconductor diode described by the Shockley diode equation (equation (1))^35^,^36^: (i) direct band-to-band recombination (as in a pn-diode, with *n*_id_=1 and *E*_μ_=*E*_g_), (ii) trap-assisted recombination based on Shockley-Read-Hall (SRH) recombination (with *n*_id_=1–2 and *E*_μ_=*E*_g_), and (iii) majority carrier emission over a barrier of height *φ*_B_ (as in the Schottky diode, with *n*_id_=1 and *E*_μ_=*φ*_B_<*E*_g_). Our finding that *n*_id_=2 and *E*_μ_≈*E*_g_ indicates that the diode current is controlled by trap-assisted recombination described by the SRH model, in agreement with the finding of Yoon *et al*.^24^ The proposal in earlier reports that the current is controlled by Schottky-type carrier transport and is determined by the metal–semiconductor interface^15^,^21^ is inconsistent with our observation that *E*_μ_≈*E*_g_ and stems from the fact that the ideality factor in equation (2) was neglected. Reanalyzing the data of ref. 15 gives *n*_id_=1.6 and *E*_μ_=0.84 *E*_g_ in agreement with our finding here that the current in NC solid MSM diodes is governed by trap-assisted recombination^37^.

From the fact that *E*_μ_=0.88 *E*_g_, we conclude that the energy states through which the mobile electron and hole travel derive directly from the quantum-confined states that give rise to lowest energy optical exciton. The discrepancy of 12% between *E*_μ_ and *E*_g_ could be due to a number of reasons such as reduced NC confinement potential after ligand exchange^38^ or bringing the NCs together in a solid^39^, or statistically higher occupation of low-energy sites in a disordered system^40^.

Our fits to the IVT data allow us to start to build a consistent picture of charge transport in PbS NC diodes, shown schematically in Fig. 2f, where free charge carriers travel through the diode at energy levels derived from the quantum-confined states of the individual NCs and experience trap-assisted recombination. We note that the symmetry of our device does not allow us to distinguish between electrons and holes. We assume that the recombination is dominated by free electrons recombining with trapped holes; however, the analysis and discussion below are equally applicable to the case where free holes recombine with trapped electrons.

To determine the physics of the trap-assisted recombination process, we consider the strong increase in the prefactor of the saturation current (*J*_00_) with *E*_g_ (log(*J*_00_)=9.15 *E*_g_−11.4), which indicates that the trap-assisted recombination becomes faster with smaller-sized (larger bandgap) NCs. To gain quantitative insight into this trend, we write the diode current (*J*_D_) by integrating the position-dependent SRH recombination rate (*U*_SRH_) over the film thickness (*d*)^35^,^36^:


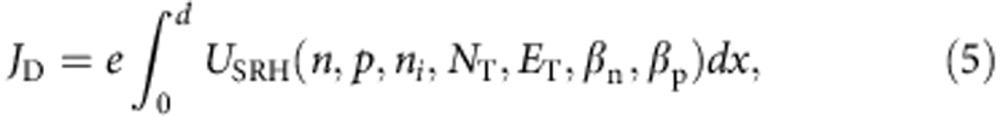


where *n* and *p* are the free electron and hole concentrations, *n*_*i*_ is the intrinsic carrier concentration, *N*_T_ is the density of traps and *β*_n_, *β*_p_ are the capture coefficients of the traps for free electrons and holes. To simplify this expression, we assume that recombination occurs homogeneously in a region of width *W*_R_, that capture coefficients for electron and holes are equal (*β*_n_=*β*_p_=*β*), and that the effective density of states of the conduction and valence bands are equal (*N*_C_=*N*_V_=*N*_CV_). A detailed derivation and analysis of all assumptions and approximations are given in the Supplementary Note 2, which allows us to approximate equation (5) by


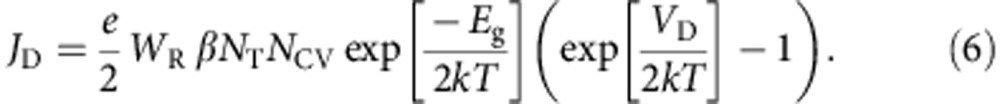


Comparing equation (6) with equations (1 and 2), we obtain *n*_id_=2, *E*_μ_=*E*_g_ and *J*_00_*T*^2^=*e*/2(*W*_R_*βN*_T_*N*_CV_). As explained in Supplementary Note 2, we assume that *W*_R_ and *N*_CV_ are independent of *E*_g_, so that we can express the slope of *J*_00_ as


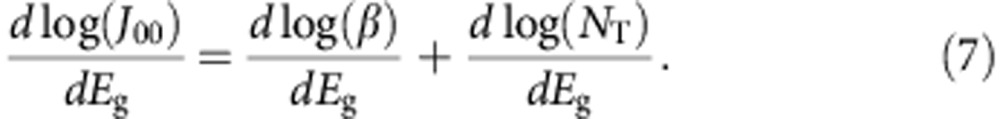


This expression enables us to assess the relative contributions of the density of trap states (*N*_T_) and the capture coefficient (*β*) to the recombination rate. Using the TAS measurements to estimate the number of traps that act as recombination centres (dlog(*N*_T_)/d*E*_g_=3.5 eV^−1^), we conclude that 38% of the increase in *J*_00_ with increasing *E*_g_ (dlog(*J*_00_)/d*E*_g_=9.15 eV^−1^) is due to an increase in the number trap states, such that the majority (62%) of the increase in *J*_00_ is due to an increased capture coefficient (*β*), which will be reflected by a trend in the capture coefficient with NC bandgap: dlog(*β*)/d*E*_g_ of 5–6 eV^−1^.

**Figure 3:**
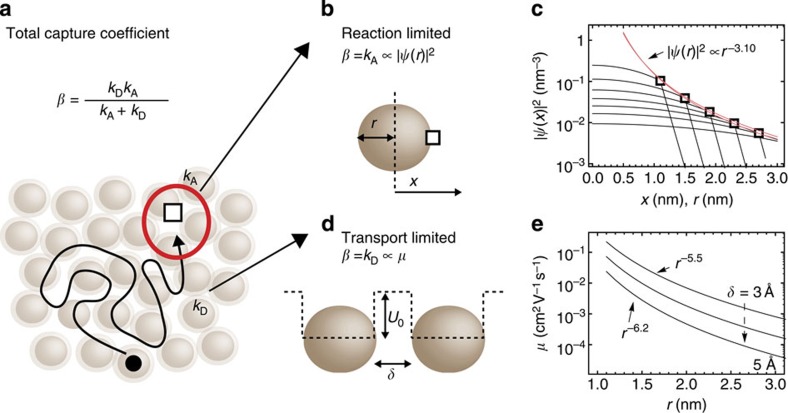
Modelling of the charge recombination process and its NC-size dependence. (**a**) The capture coefficient (*β*) of recombination can be modelled as a combination of diffusion (*k*_D_) and reaction (*k*_A_). (**b**) In the reaction limited case (*k*_A_≪*k*_D_), *β* is assumed to be proportional to the electron wavefunction at the surface of the NC (|*ψ*(*r*)|^2^). (**c**) Calculating the electron wavefunction, |*ψ*(*x*)|^2^, in individual NCs of different sizes (black lines) shows that the wavefunction at the surface of the NC scales as |*ψ*(*r*)|^2^∝*r*^−3.10^ (red line). (**d**) In the diffusion-controlled case (*k*_A_≫*k*_D_), *β* is given by equation (9) and is proportional to the charge carrier mobility (*μ*). (**e**) Calculation of *μ* in dependence of NC radius (*r*) and inter-NC distance (*δ*) based on ref. 39. *μ* scales according to *r*^−5.5^ to *r*^−6.2^.

We now assess what physical process for recombination can explain the increase in capture coefficient with increasing NC bandgap given by dlog(*β*)/d*E*_g_ of 5–6 eV^−1^. As shown schematically in Fig. 3a, the recombination of a free charge carrier with a trapped charge carrier of the opposite polarity can be described as a combination of (i) a diffusion process with a coefficient (*k*_D_) and (ii) a reaction process with a coefficient (*k*_A_). The total capture coefficient is given by the reaction kinetics as^41^:


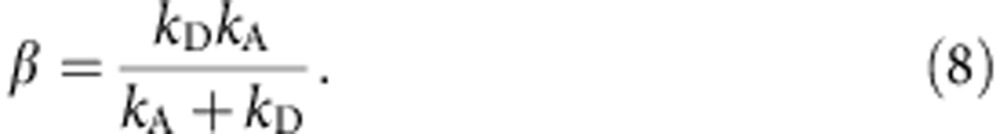


We consider the two limiting cases, where either the reaction or the diffusion dominates the total rate. In the first case, where recombination is controlled by the reaction between a free carrier and a trapped carrier (*k*_A_≪*k*_D_; Fig. 3b): *β*=*k*_A_. The coefficient *k*_A_ is determined by the matrix element coupling the free charge carrier to the trap state (*k*_A_∝|*M*_if_|^2^). To estimate how the matrix element changes with NC size, we assume that it is proportional to the wavefunction (*ψ*) of the free carrier at the surface of the NC (that is, *k*_A_∝|*ψ*(*r*)|^2^). We calculate the wavefunction of the confined electron in the NC based on equations (12–17) from ref. 39 and plot |*ψ*(*x*)|^2^ in Fig. 3c for NC radii from 1 to 3 nm. The wavefunction at the surface of the NC is found to scale approximately as the inverse NC volume (|*ψ*(*r*)|^2^∝*r*^−3.1^). To compare this scaling (*β*∝*r*^−3.1^) to the measured change in *β* versus *E*_g_, we use ref. 27 to relate the radius to the bandgap (*E*_g_=0.41+0.85 *r*^−1^+0.96 *r*^−2^) and fit a linear function to the calculated log(*β*(*E*_g_)). We obtain a trend with NC bandgap (dlog(*β*)/d*E*_g_=2.66 eV^−1^) significantly smaller than that expected from our measurements (dlog(*β*)/d*E*_g_ of 5–6 eV^−1^), and therefore conclude that recombination in NC solids is not primarily determined by the reaction coefficient of a trapped carrier.

In the second case, where the recombination is controlled by diffusion (*k*_A_>>*k*_D_), the capture coefficient is given by *β*=*k*_D_ (Fig. 3d). We model the trapped charge, to which a free charge carrier must diffuse to recombine, as a sphere of radius (R*). Solving the diffusion equation, we obtain:


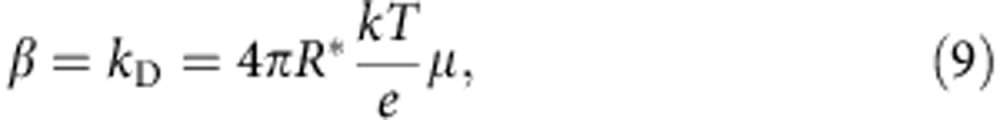


where *μ* is the charge carrier mobility^41^. Using equations (18–20) from ref. 39, we calculate *μ* in dependence of the NC radius for different spacings between the NCs (*δ*=3–5 Å). The results are plotted in Fig. 3e and depending on the inter-NC distance, the mobility scales as *r*^−5.5^ to *r*^−6.2^. This increase in mobility with decreasing NC size has recently been shown experimentally using time-of-flight measurements^42^ and can be understood by the fact that decreasing NC size leading to an increased charge carrier energy and a decrease in the effective energy barrier between NCs, such that more of the wavefunction leaks out of the NC and improves the electronic coupling between NCs. In the Supplementary Note 3, we discuss these experimental and theoretical findings in relation to previous studies on the mobility in field effect transistors. Relating the NC radius to *E*_g_, we find that the dependence of the capture coefficient on NC bandgap assuming a diffusion-controlled recombination process is dlog(*β*)/d*E*_g_=4.9–5.4 eV^−1^. Returning to equation (7), a diffusion-controlled recombination process thus explains the experimentally observed increase in prefactor of the saturation current with NC bandgap (dlog(*J*_00_)/d*E*_g_=9.15 eV^−1^) that is not explained by the increase in trap-state density with increasing NC bandgap (dlog(*Ν*_T_)/d*E*_g_=3.5 eV^−1^).

In summary, the IVT and TAS characterization show that charge carriers in NC solids travel in states derived from the energy levels of the NCs and that the current is govern by trap-assisted recombination. This recombination process is controlled by diffusion of free charge carriers to trap states, which explains why recombination increases with decreasing NC size: NC solids composed of smaller-sized NCs have more trap states and exhibit higher mobility because of increased electronic coupling between neighbouring NCs.

### Origins of the series resistance

At voltages greater than 1 V, the IV curves flatten out and are consistently described by a series resistance of the form in equations (3 and 4), indicative of a barrier-lowering process. To fit the data over all temperatures, we only need to adjust two parameters (*E*_rs_ and *R*_s00_); the third parameter *α* is fixed for all devices. Two physical processes with the form of equations (3 and 4) are the: (i) Schottky effect and (ii) Poole-Frenkel effect^29^. Our IVT and TAS characterization as a function of NC size enables us to determine which of these two processes most accurately describes the series resistance in the MSM diodes.

The Schottky effect describes the thermionic emission of electrons from a metal electrode over an energy barrier into a semiconductor. The current that can be supplied by this effect is at least *A*_0_*T*^2^exp(−*E*_rs_/*kT*), where *A*_0_=120 A cm^−2^ K^−2^ is the Richardson constant and *E*_rs_ the barrier height^29^. For the device shown in Fig. 1a, for example, *E*_rs_=0.35 eV, so the Schottky effect could supply a current of at least 9 × 10^3^ mA cm^−2^, which is significantly larger than the observed current (~10^2^ mA cm^−2^). We conclude therefore that the Schottky effect cannot quantitatively explain the limited currents in the diode at large forward bias.

The PF effect is the lowering of the electrostatic barrier, when a trapped charge carrier leaves a trap state^43^. In the past, it has been used to describe the bulk conductivity in defect-rich and compensated semiconductors^44^, and, more recently, in chalcogenide glasses^45^. The bulk conductivity in the PF model is given in dependence of the electric field (*F*) and the temperature (*T*) by


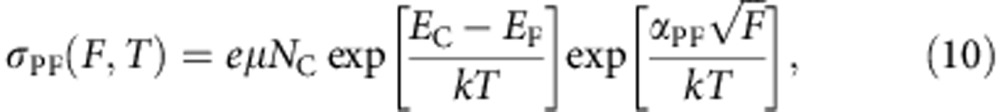


where *E*_C_ is the conduction band energy and the barrier-lowering constant 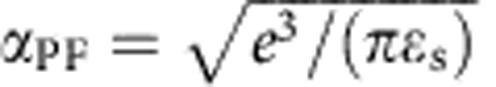. For our PbS NC solid, we take a dielectric constant, *ε*_s_=15 *ε*_0_ (ref. 37), so that *α*=1.96 × 10^−4^ eV V^−1/2^ cm^1/2^, which we have used in equation (3) for all fits of the IVT characteristics. Relating the series resistance from equations (3 and 4) to the PF conductivity from equation (10) by setting *R*^−1^_s_=*σ*_PF_/*d* and *F*=*V*_D_/*d*, we find that the activation energy of the series resistance is given by the position of the Fermi-level (*E*_rs_=*E*_C_−*E*_F_) and the prefactor of the series resistance is given by


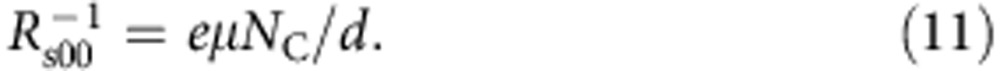


Since the prefactor *R*^−1^_s00_ is proportional to the charge carrier mobility, it should show the same trend with NC bandgap as the mobility. Indeed, using equation (11) and the calculated mobility from Fig. 3e, and assuming *N*_C_=10^19^ cm^−3^ and *d*=100 nm, we plot *R*^−1^_s00_ (shaded region in Fig. 2b) and find good quantitative agreement with the measured values.

The TAS measurements quantitatively support our conclusion that the series resistance stems from the PF effect. When PF conduction occurs in a semiconductor, the Fermi-level is expected to be pinned by the trap states close to the trap-state energy^29^. The fact that the TAS response is non-resonant (*E*_TF_>0) also indicates a pinned Fermi-level^31^. Indeed, comparing Fig. 3a,c, we find that the activation energy of the conductivity (*E*_rs_=*E*_C_−*E*_F_) and the trap activation energy (*E*_T_) are close in magnitude and follow the same trend with NC bandgap.

The attribution of the PF effect to the series resistance also gives insight into the type of trap states present in the PbS NC solid. Barrier lowering in the PF model requires that the trap states be charged when unoccupied. This is consistent, for example, with a donor-type trap close to the conduction band as shown in Fig. 2f. Such donor-type states are predicted below the conduction band by density functional theory (DFT) calculations in the case of a small excess (2%) of lead in comparison to sulphur^34^.

Our analysis shows that the observed series resistance does not stem from charge injection of the metal to the semiconductor. The metal is able to inject a much larger current into the semiconductor than we observe and can therefore not be limiting the current. Instead, PF conduction occurring in the bulk of the NC solid is confirmed by the quantitative agreement of the activation energy (*E*_rs_), the prefactor (*R*_s00_) and the barrier-lowering constant (*α*) with the theoretical predictions of the PF-model and the trap-state measurements (TAS).

### General applicability of model

Our analysis shows that the charge carrier transport mechanisms governing our devices derive from the properties of NC solids, rather than from the device architecture (that is, interfaces or contacts). This implies that our charge transport model should apply equally well to other device architectures and NC solids. We confirm the generality of our model by characterizing a heterojunction device (TiO_2_/PbS:EDT/MoOx/Au) and a MSM device using 1,4-benzenedithiol as a crosslinker. Data presented in the Supplementary Note 4 show that both devices reproduce the main findings presented above: (i) *n*_id_ between 1.6 and 2, (ii) *E*_μ_~0.8 *E*_g_, (iii) a thermally activated series resistance (*E*_rs_) showing barrier-lowering as described by equations (3), (iv) *R*_s00_ in agreement with values shown in Fig. 2b, and (v) a trap energy (*E*_T_) matching *E*_rs_ in reasonable agreement with Fig. 2a,c. These results underscore that our model successfully captures the key elements of charge transport in NC solids, independent of device architecture or the ligand host. For the convenience of the reader, we have summarized all model equations in Supplementary Note 5.

### Non-trivial dependence of diode parameters on NC size

**Figure 4:**
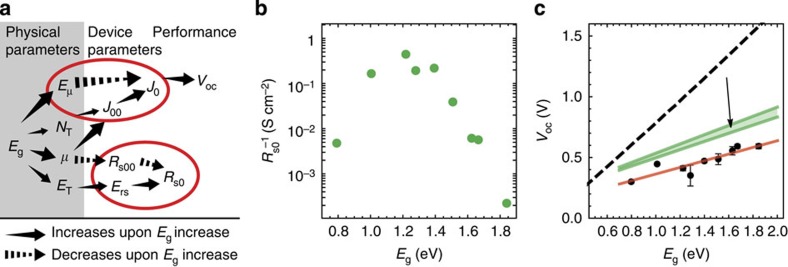
The influence of correlation and compensation in charge transport parameters. (**a**) Schematic of correlations determined by this study. Solid arrows indicate an increase of the target parameter upon an increase of optical bandgap (*E*_g_). Dashed arrows indicate a decrease. Parameters, which are targeted by both a solid and dashed arrow (indicated with red circles), are subject to the Meyer–Neldel rule. (**b**) Series resistance at 300 K shows non-monotonic trend, because of compensation of *R*_s00_ and *E*_rs_. (**c**) Open-circuit voltage (*V*_oc_) as a function of *E*_g_ (black squares) with a linear fit to the data (red). *V*_oc_-limit imposed by the dark current (equation (12)) is shaded in green for temperatures between 290 and 315 K. Ideal thermodynamic limit is indicated as a reference (dashed line). Error bars indicate the standard deviation over the measurement of eight equivalent devices.

One of the major appeals of NC solids is the possibility to tune their bandgap by the size of the constituent NCs. However, in addition to the bandgap varying with NC size, the free carrier mobility (*μ*), the number density of trap states (*N*_T_) and the energetic depth of the trap states (*E*_T_) also vary with NC size. As depicted in Fig. 4a, these parameters in turn play a role in the diode saturation current (*J*_0_) and the series resistance (*R*_s_), which describe the current in a NC solid. When the effect of two parameters compensate, one finds non-trivial dependence of charge transport on NC size as shown for the series resistance (*R*_s0_) in Fig. 4b. The red circles in Fig. 4a highlight that compensation occurs for both the diode saturation current (*J*_0_) and the series resistance (*R*_s_). The physical origin for this compensation is the exponential increase of the free carrier mobility (*μ*) with decreasing NC size and the increase in activation energies with increasing NC bandgap (that is, decreasing NC size). The prefactors (*J*_00_ and *R*^−1^_s00_) are both proportional to the free carrier mobility and compensate in part for the activation energies (*E*_μ_ and *E*_rs_), which increase with decreasing NC size and appear in exponent of the expression for *J*_0_ and series resistance *R*_s_ (equations 2 and 3). Rather than being an exception, such compensation is known as the Meyer–Neldel rule^25^ and is to be expected for disordered systems, such as NC solids. For an engineer trying to develop a higher-performance NC-based device, it is critical to determine the impact of a chemical procedure on the charge transport in the solar cell correctly. Our work shows that temperature-dependent measurements are necessary to distinguish between the changes of activation energies and prefactors and that they alleviate the challenges in understanding posed by compensating parameters in the expressions for charge transport.

## Discussion

Here we discuss the implications of our findings for NC-based solar cells and use insights from our model to develop design guidelines and propose realistic fabrication strategies to achieve higher power conversion efficiencies in NC-based solar cells. Specifically, we describe the trade-off between open-circuit voltage and short circuit current stemming from size-dependent charge transport properties. We then use our model to explain why (i) balancing of electron and hole mobilities and (ii) controlling these mobilities and trap states independent of NC-size are key to minimizing recombination while improving charge extraction. We describe how judicious ligand selection and control of NC composition could be used to implement these design rules and achieve high-performance NC solar cells.

From thermodynamic considerations, an engineer who increases the bandgap of a NC solar cell expects that the open-circuit voltage (*V*_oc_) will increase by about the same amount (dashed line in Fig. 4c). However, the open-circuit voltages from our devices (data points in Fig. 4c) fall consistently below this line in good agreement with earlier reports^22^,^23^,^24^,^46^,^47^. Our dark current measurements give us insight into the origins of this *V*_oc_ deficit and its increase for higher bandgaps. Using the Shockley diode equation (equation (1)), we can derive a strict upper limit for the open-circuit voltage (*V*_oc_) set by the diode current^20^:


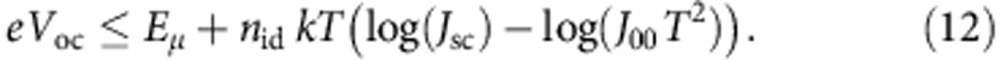


This upper limit is plotted in Fig. 4c by using the fits derived from our measurements (Table 1), and shows a strong *V*_oc_ deficit, similar to that in the measured *V*_oc_. Our results explain that the moderate slope of this *V*_oc_ limit, and accordingly the *V*_oc_ deficit, do not result from a deficiency of the mobility bandgap (*E*_μ_), which increases like the optical bandgap (*E*_μ_=0.88*E*_g_). Instead, the *V*_oc_-deficit results predominantly from the increase in *J*_00_, as a result of the increase of both the number of trap states and charge carrier mobility with decreasing NC size. Although the limit of equation (12) does not fully explain our observed *V*_oc_ deficit, it provides a better understanding of the origins of the *V*_oc_ deficit than previously established^24^ and indicates that the slope of the *V*_oc_ versus *E*_g_ is likely limited by intrinsic compensation effects.

Although a higher free carrier mobility with increasing NC bandgap (decreasing NC size) means that free charge carriers will more quickly find recombination centres, increasing the *V*_oc_ deficit, this increase in mobility improves charge extraction and therefore the short circuit current (*J*_sc_). This trade-off between *J*_sc_ and *V*_oc_ with mobility has previously been discussed in the case of organic solar cells, which in analogy with our NC solids feature mobility-dependent recombination rates^36^,^48^, and successfully explains why NC solids of intermediate charge carrier mobilities (10^−3^ to 10^−2^ cm^2^ V^−1^ s^−1^)—not with those with highest mobilities (1–10 cm^2^ V^−1^ s^−1^)—result in the solar cells with the best power conversion efficiencies^49^. In this context, the recently described ‘mobility-invariant regime’ of PbS NC solar cells has to be restricted in its interpretation to a saturation of charge extraction^9^: the open-circuit voltage will decrease for higher mobilities in a semiconductor, such as a NC solid, where recombination is controlled by diffusion^49^.

This understanding of the complex interdependences influencing charge transport and solar cell performance enable us to present design guidelines for achieving high-performance NC-based solar cells and suggest practical methods to implement these guidelines. First, our finding of a diffusion-limited recombination process, means that recombination is controlled by the diffusion of the faster species. The design rule that follows from this finding is that charge carrier mobilities must be balanced to minimize recombination. For the case of the alkane dithiols, it was shown that the ratio of electron and hole mobilities can easily exceed 3, which may explain why alkane dithiols do not yield record efficiencies^50^. Recent work has shown that the surface dipoles induced by the ligands have a large impact on charge transport^14^. Such surface dipoles should increase the tunnel barriers for one type of carrier, while decreasing the barrier for the other carrier, thereby presenting an opportunity to control the ratio between the electron and hole mobilities. Selection of ligands to control interface dipoles to create symmetric electron and hole mobilities can thus be used to improve device efficiencies.

Second, to leverage the feature of a bandgap that is tunable with NC size, it is furthermore critical to be able to adjust the free charge carrier mobility and the pinning of the Fermi-level independently. Previous work has shown that the mobility depends strongly on the distance between NCs (*δ*): *μ* ∝ exp(−*βδ*), with *β*~1.1 Å^−1^. From an engineering perspective, the size dependence of the free charge carrier mobility can be adjusted by using ligands of different lengths^50^. We further showed bandgap-dependent pinning of the Fermi-level by trap states. This effect is in most cases undesired for charge extraction, in particular if it pins the Fermi-level energetically far away from valence or conduction band and spatially far away from the electrodes. It was recently shown that the Fermi-level in NC solids can be adjusted through the stoichiometry of the material^34^,^51^ or doping^52^. If the pinning of the Fermi-level can be controlled, it can be useful to create ohmic contacts through highly doped n-type (n^−−^) or p-type (p^++^) regions close to the electrodes^2^.

In conclusion, we performed IVT measurements and TAS on a series of NC-based MSM diodes with optical bandgaps (*E*_g_) from 0.79 to 1.85 eV. We found that changing NC *E*_g_ directly translates into an equivalent increase of the mobility bandgap (*E*_μ_), confirming the long held assumption that charge carriers are transported in states that derive from the quantum-confined electronic levels of the individual NCs. The diode current results from trap-assisted recombination of electrons and holes in the diode, controlled by the diffusion of the faster charge carrier to a trapped charge of the opposite polarity. At high forward bias, the diode current is limited by a series resistance that is due to trap states, which pin the Fermi-level between 0.1 and 0.4 eV away from the conduction band, depending on the constituent NC size. Our results enabled us to disentangle the different physical quantities that influence charge transport and propose a model for charge transport in NC solids, which we demonstrate to be general by showing its applicability to different NC-based devices architectures and NC solids. In the discussion, we used our model to propose design guidelines to achieve higher power conversion efficiencies in NC-based solar cells and realistic fabrication strategies. This quantitative model for the charge transport in NC solids can further be used in conjunction with characterization techniques to systematically explain how novel NC chemistries, surface treatments and fabrication techniques influence charge transport parameters and can be effectively leveraged to achieve high-performance NC-based devices.

## Methods

### NC synthesis and characterization

The PbS NCs were synthesized using the procedure described by Hines *et al*.^26^ and Choi *et al*.^53^ In a typical synthesis of 3 nm PbS NCs, 1.8 g of PbO was mixed with 5 ml of oleic acid and 75 ml of 1-octadecene in a three-neck flask. The mixture was refluxed at 150 °C for 2 h, during which lead (II) oleate was formed. Then, the reaction flask was filled with nitrogen, and 40 ml of 0.1 M solution of hexamethyldisilathiane in pre-purified octadecene was swiftly added. The reaction mixture was cooled to 100 °C and kept at this temperature for another 5 min. Finally, the reaction was terminated with a water bath and the obtained PbS NCs were purified by a standard solvent/nonsolvent procedure, using hexane and ethanol. The mean size of the PbS NCs was controlled by the amount of oleic acid and the injection temperature, as described in refs 26, 53. TEM images shown in Supplementary Fig. 1 were acquired with a Philips CM12 electron microscope operating at 100 kV.

### Device fabrication and characterization

All measurements were performed on devices fabricated as described in refs 18, 32 with an active area of 2.0 mm^2^. A PbS NC-layer was deposited by sequential dip-coating in a PbS NC solution (5 mg ml^−1^ in hexane), a crosslinking solution (6 mM EDT in acetonitrile) and a rinsing solution (acetonitrile). The crosslinking and rinsing solutions use anhydrous acetonitrile, stored and prepared in an N_2_ glovebox, and brought into ambient immediately before dip-coating. Dip-coating was carried out in air. The number of layers dip-coated was adjusted between 23 and 35 to achieve a film thickness of 100 nm, determined by atomic force microscopy. On top, we evaporated a LiF(1 nm)/Al(100 nm)/Ag(250 nm) electrode in high vacuum, starting at a base pressure of 3 × 10^−7^ mbar and reaching a maximum pressure of 7 × 10^−7^ mbar during evaporation.

After a short air exposure of 5 min, the sample was mounted into a cryostat (Janis ST-500), where it remained in vacuum during the IV measurements. Each sample has eight devices. First, we performed an IV measurement in the dark on all devices, sweeping the bias voltage from −1 V (reverse) to +2 V (forward) back to 1 V at a rate of 80 mV s^−1^. Under AM1.5G illumination (Oriel Arc Lamp F/1 with AM1.5G filter), we repeated the same measurement to determine the solar cell performance of each of the eight devices. Based on the dark and light IV measurements of the eight devices, a representative device was chosen for temperature-dependent IV measurements.

### Temperature-dependent IV measurements (IVT)

IVT measurements were performed directly after device fabrication and the solar cell characterization. For the measurement, we swept the bias voltage continuously from −1 V to +2 V and back at a rate of 80 mV s^−1^. In parallel, we ramped the temperature from room temperature down to ~150 K at a constant rate of 5 K min^−1^. Voltage, current and temperature (measured at the device) were recorded every 0.5 s.

### TAS measurements

TAS measurements were performed after storing the sample for 2 days in ambient. For each TAS measurement, we measured the real and imaginary part of the capacitance with an impedance analyser (Solartron MODULAB MTS) for frequencies of 10 Hz–1 MHz at 0 V bias using a modulation amplitude of 10 mV. This measurement was performed continuously while ramping the temperature from 310 K down to 160 K at a rate of 5 K min^−1^. The temperature at the sample was recorded for each capacitance value.

The data were analysed according to the method by Walter *et al*.^30^ For each measurement, we observed a discrete trap level. The trap activation energy was determined from an Arrhenius plot. The trap-state density was obtained by integrating the spectral trap-state density over the dominant discrete trap state^18^. Since we are in the non-resonant condition,^31^ where the trap-state energy and the Fermi-level do not coincide, we determine the distance between the Fermi-level and trap level and also used this value to compensate for the underestimation of the trap-state density as described in ref. 31.

### Analytical calculations

To calculate the wavefunction of an electron in a single NC and the electron mobility of a NC solid, we used equations (12–21) from ref. 39 with the following parameter values (and ranges): a barrier potential of *U*_0_=1.6 eV (ref. 50) a disorder parameter *χ*=0.1; an inter-NC distance of *δ*=*b*–2*a*=3–5 Å; and an electron effective mass in the barrier *m*=*m*_e0_, where *m*_e0_ is the free electron mass. For the electron effective mass in the PbS NC (m*), we used the non-parabolic approximation for PbSe given in ref. 39: *m*/m*=3.9+2.64/(*E*_0_+0.28), where *E*_0_ is the ground-state energy of the quantum-confined electron. We verified our calculations by reproducing the results of Figs 2a,c,e and 3a in ref. 39.

## Author contributions

D.B. and W.M.M.L. performed the experiments and analysed the data, D.B. performed calculations and modelling, N.Y. performed heterojunction experiments, O.Y. synthesized and characterized the materials, D.B. and V.W. devised the experiments. All authors contributed to the writing of the manuscript.

## Additional information

**How to cite this article:** Bozyigit, D. *et al*. Demystifying charge carrier transport, trapping and recombination in nanocrystal-based solar cells. *Nat. Commun.* 6:6180 doi: 10.1038/ncomms7180 (2015).

## Supplementary Material

Supplementary Information Supplementary Figures 1-11, Supplementary Tables 1-4, Supplementary Notes 1-5, and Supplementary References

## Figures and Tables

**Table 1:** Bandgap dependence of charge transport parameters.

**Method**	**Parameter**	**Fit**	**Unit**	**Description**
Optical absorption	*E*_g_	=0.41+0.85*r*^−1^+ 0.96*r*^−2^ (ref. 27)	(eV)	Optical bandgap
Light IV	*V*_oc_	=0.27 *E*_g_+0.09	(V)	Open-circuit voltage
	*J*_sc_	=−4.28 *E*_g_+20.1	(mA cm^−2^)	Short-circuit current
Dark IVT	*n*_id_	=2.13±0.23	(1)	Ideality factor
	*E*_μ_	=0.88 *E*_g_	(eV)	Mobility bandgap
	Log(*J*_00_)	=9.15 *E*_g_−11.4	Log(mA cm^−2 ^K^−2^)	Red. saturation current
	*E*_rs_	=0.29 *E*_g_−0.16	(eV)	Series res. act. energy
	Log(*R*^−1^_s00_)	=6.23 *E*_g_−1.75	Log(Scm^−2^)	Series res. prefactor
TAS	*E*_T_	=0.43 *E*_g_−0.31	(eV)	Trap activation energy
	*E*_TF_	=0.14 *E*_g_−0.15	(eV)	Fermi to trap energy
	Log(*N*_T_)	=3.49 *E*_g_+37.1	Log(cm^−3^)	Trap-state density

Act, activation; IV, current–voltage characterization; IVT, temperature-dependent current–voltage; red, reduced; res, resistance; TAS, thermal admittance spectroscopy.

Linear fits as function of the optical bandgap (*E*_g_) for parameters determined by current–voltage (IV) characterization in light, temperature-dependent IV characterization in dark and thermal admittance spectroscopy (TAS) measurements. The NC radius (*r* in nm) is determined from *E*_g_ based on the relation in the first row^27^.
