# Supplementary material for: A quantitative model for charge carrier transport, trapping and recombination in nanocrystal-based solar cells
Source: Nat Commun. 2015 Jan 27;6:6180. doi: 10.1038/ncomms7180 (PMC4317500; doi:10.1038/ncomms7180)
Supplement: Supplementary Information — Supplementary Figures 1-11, Supplementary Tables 1-4, Supplementary Notes 1-5, and Supplementary References [file ncomms7180-s1.pdf]

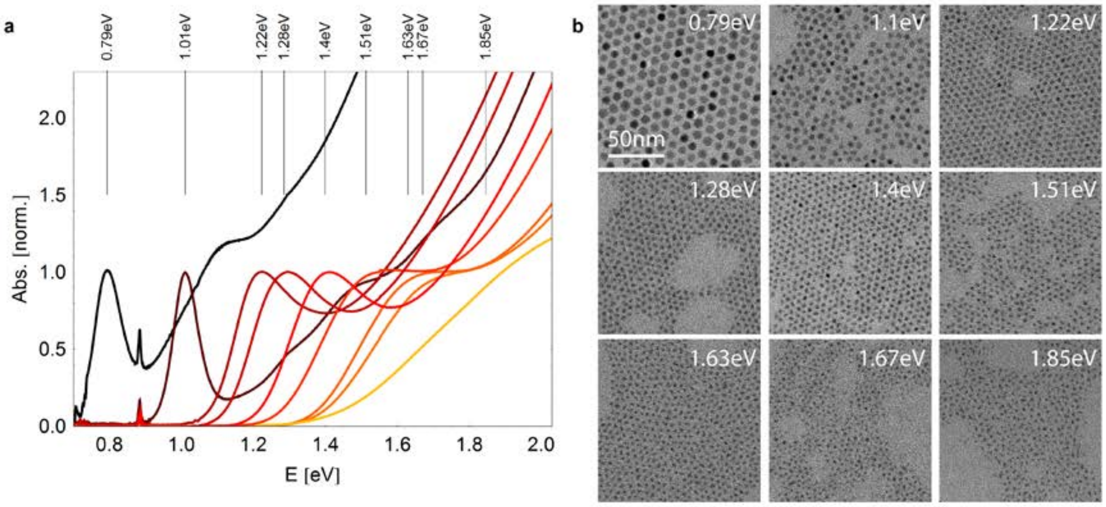

Supplementary Figure 1: Material characterization of 9 batches of nanocrystals with radii between 1.17 nm and 3 nm. (**a**) Optical absorption spectra measured with a Cary 5000 UV-Vis-NIR. We define the optical band gap (E_g) to be the peak of the lowest energy exciton. (**b**) Transmission electron microscopy images of all 9 sizes of nanocrystals labelled by their corresponding band gap E_g.

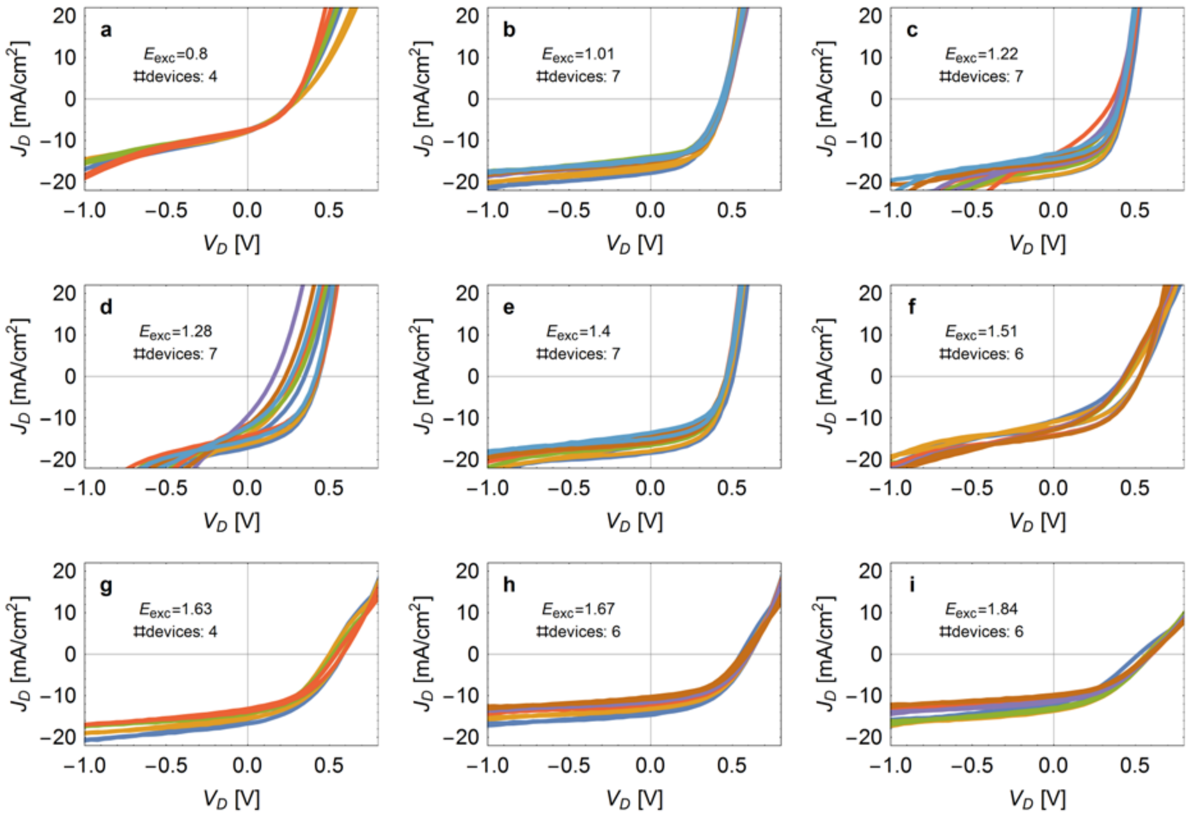

Supplementary Figure 2: Solar cell current-voltage characteristics of MSM devices under AM1.5G illumination. NC band gap (E_g) increases from **a** to **i**. Each plot provides the E_g of the constituent NCs (in eV) and the number of devices used for statistics.

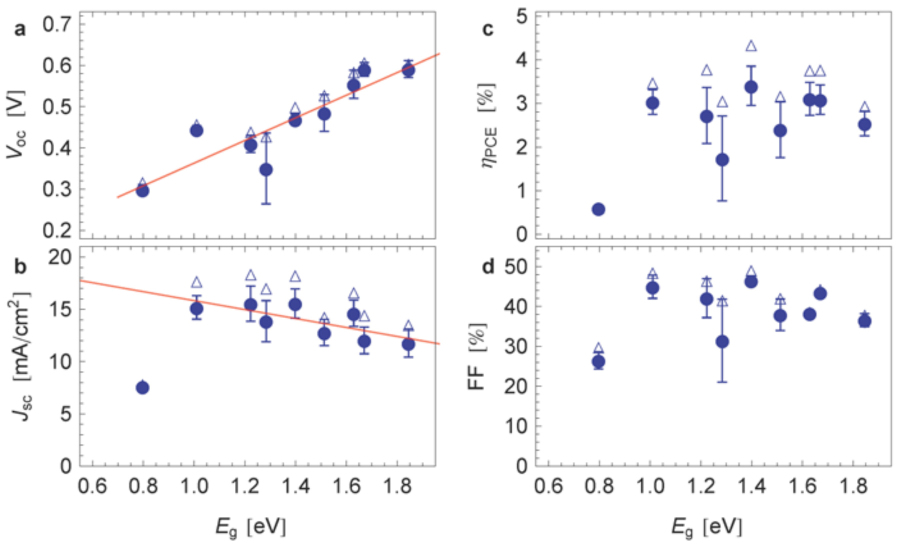

Supplementary Figure 3: Solar cell performance parameters under AM1.5G illumination. Plots show the (**a**) open-circuit voltage (V_{oc}), (**b**) short-circuit current (J_{sc}), (**c**) power conversion efficiency (η_{PCE}), and (**d**) fill factor (*FF*) for the mean (dot), standard deviation (error bars), and the best device (empty triangle) of 8 equivalent devices for each optical band gap.

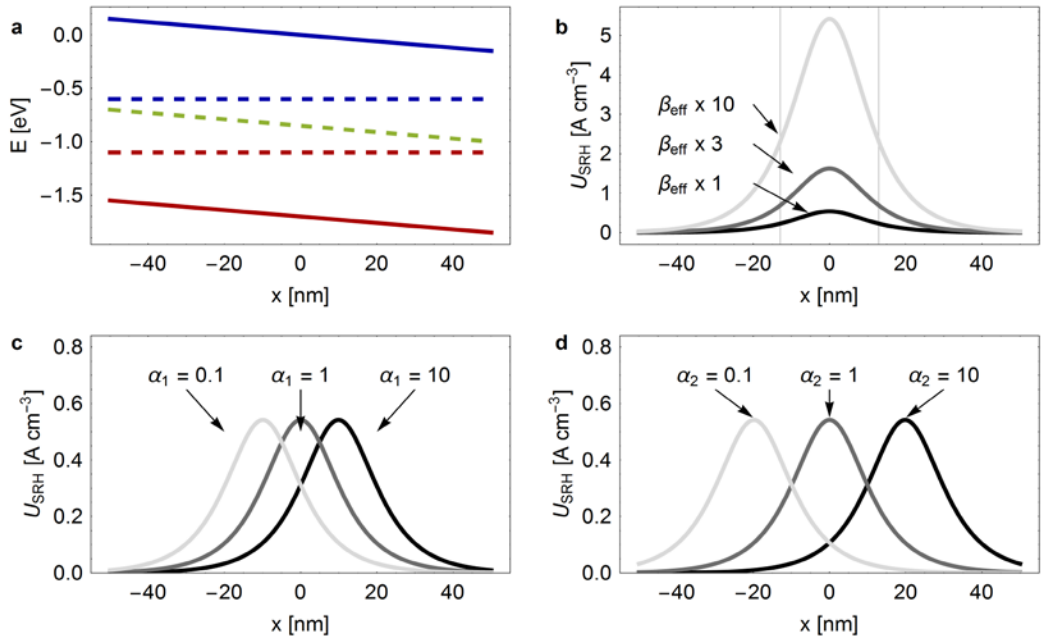

Supplementary Figure 4: Shockley-Read-Hall recombination in MSM devices. (**a**) Band diagram showing the conduction band (blue), the valence band (red), the quasi-Fermi-level for electrons (dashed blue), the quasi-Fermi level for holes (dashed red), and the intrinsic Fermi-level (dashed yellow). Recombination rate ($U_{SRH}(x)$) for different values of (**b**) β, (**c**) α_1, and (**d**) α_2. As these parameters vary, the spatial spread of the recombination rate remains the constant, indicating that the width of the recombination region is independent of β, α_1, and α_2.

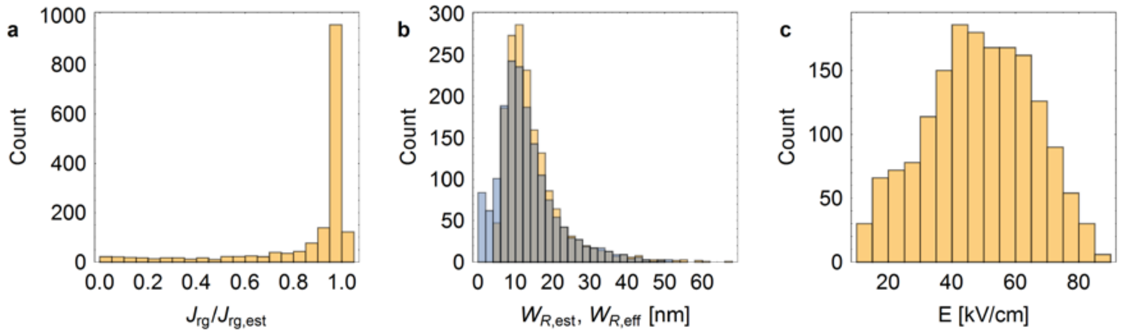

Supplementary Figure 5: Statistical analysis of the validity of the analytical approximation. (**a**) Histogram of the ratio of the numerically calculated current J_{RG} (Supplementary Equation 3) and the analytical approximation $J_{RG,est}$ (Supplementary Equation 5) (**b**) Histograms of the numerically calculated W_R (blue) and the estimated $W_{R,est}$ (Supplementary Equation 4) (orange) (**c**) Histogram of the electric field found in the calculation.

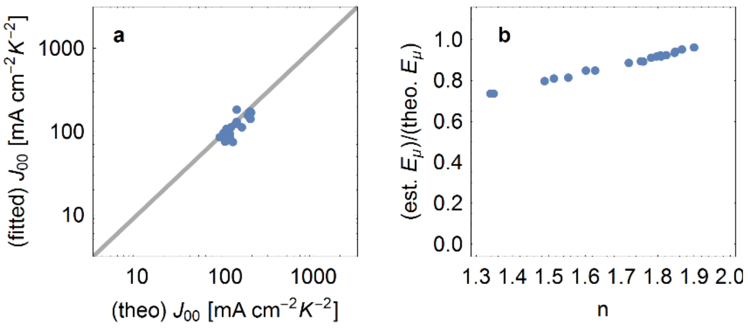

Supplementary Figure 6: Test of the parameter estimation by fitting to simulated data. (**a**) J_{00} estimated by fitting vs. J_{00} that was used for the simulation. Gray line shows identity. (**b**) Ratio of estimated band gap (est. E_μ) and value used for simulation (theo. E_μ) vs. the ideality factor (n_{id}) that was extracted by fitting. For $n_{id} < 2$ the band gap is underestimated (blue).

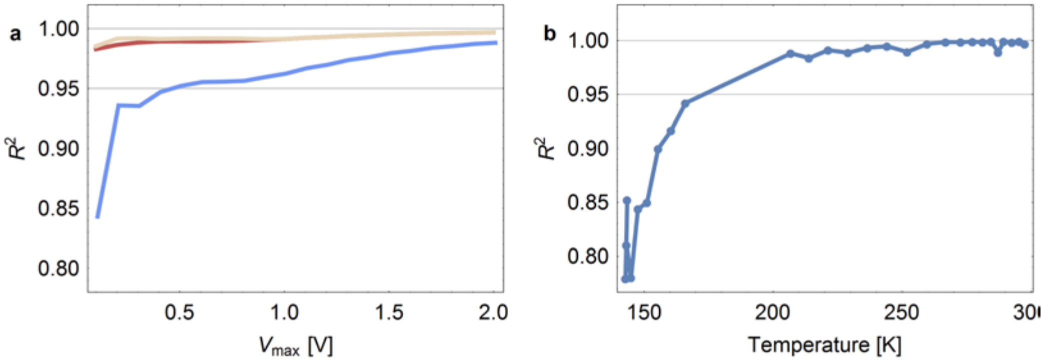

Supplementary Figure 7: Statistical analysis for the fitting of temperature-dependent current-voltage (IVT) data. (**a**) Coefficient of determination (R^2) for different voltage ranges {0, V_{max}} and temperatures (blue: 200K, orange: 250K, red: 300K) (**b**) R^2 for different temperatures.

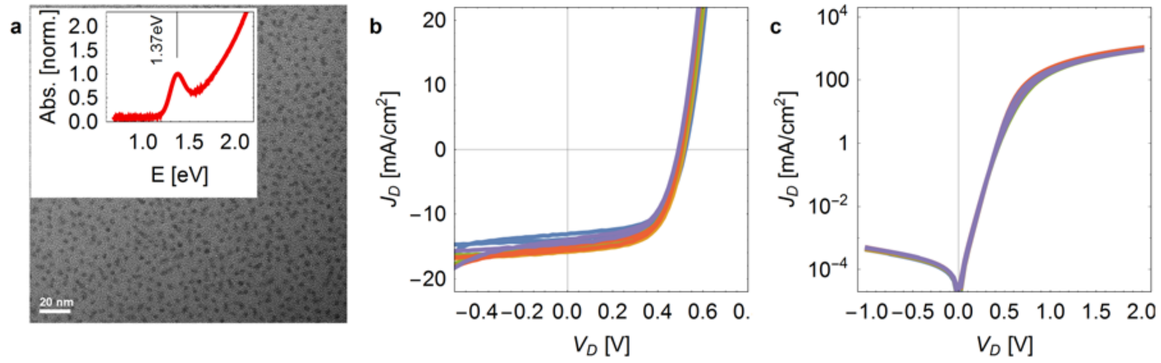

Supplementary Figure 8: Basic characterization of heterojunction device. (**a**) Optical absorption spectrum (with exciton peak at E_g=1.37eV) and TEM image of constituent NCs. (**b**) Current-voltage measurement under AM1.5G illumination and (**c**) in the dark.

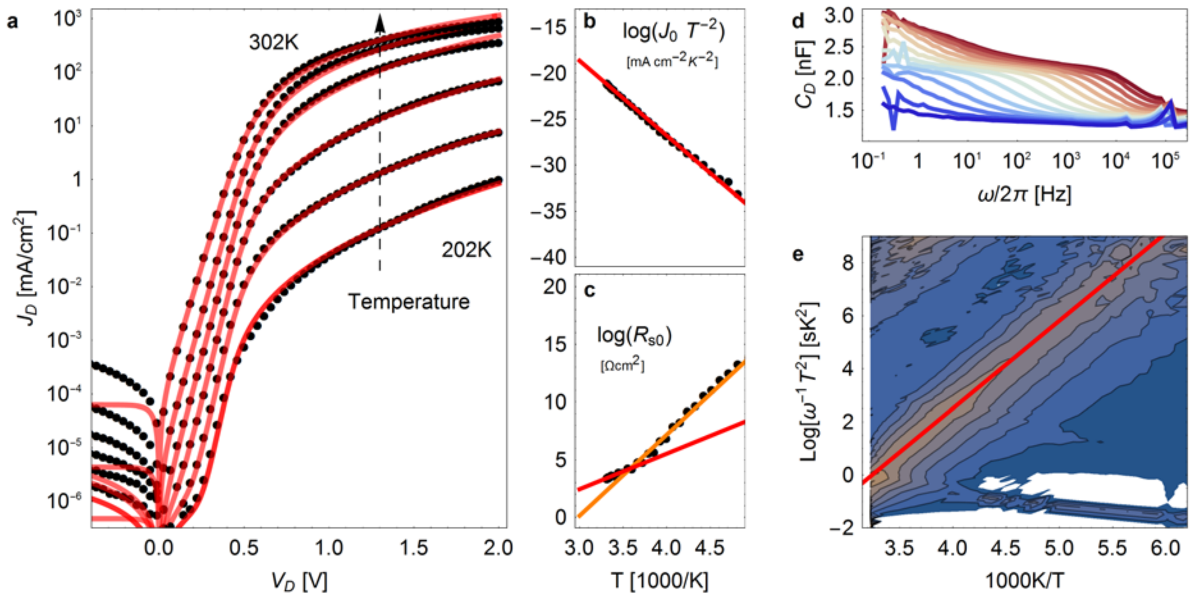

Supplementary Figure 9: Temperature dependent characterization of heterojunction device **(a-c)** Temperature dependent current-voltage (IVT) and **(d, e)** Thermal Admittance Spectroscopy (TAS). All data is extracted and plotted as explained in the main text.

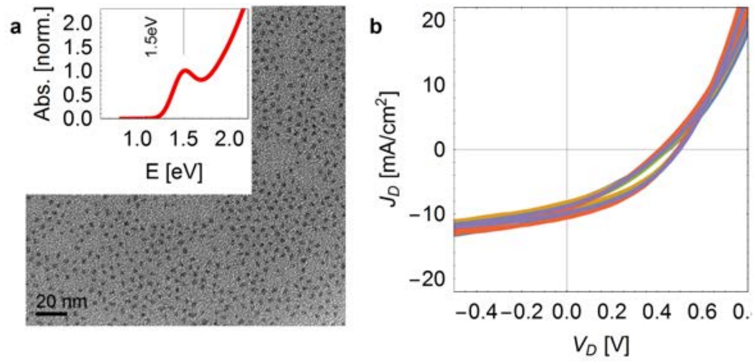

Supplementary Figure 10: Basic characterization of BDT-device. (**a**) Optical absorption spectrum (with exciton peak at E_g=1.50eV) and TEM image. (**b**) Current-voltage measurement under AM1.5G illumination.

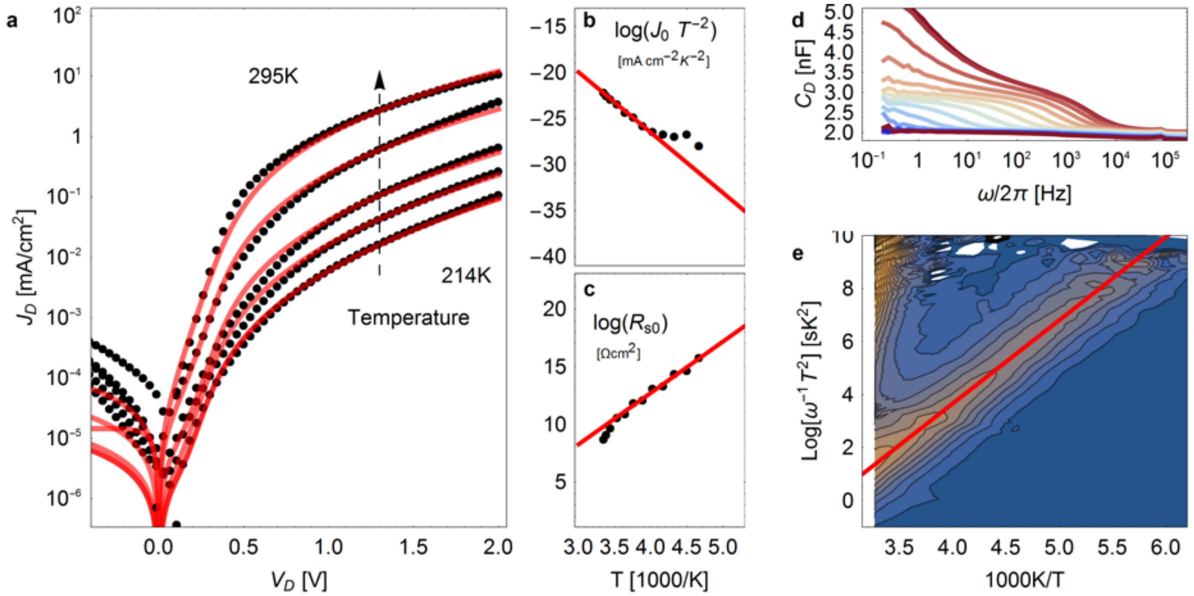

Supplementary Figure 11: Temperature dependent characterization of BDT-device (a-c) Temperature dependent current-voltage (IVT) and **(d, e)** Thermal Admittance Spectroscopy (TAS) measurements. All data is extracted and plotted as explained in the main text.

**Parameter (Unit)**	**Value**	**Explanation**
E_μ (eV)	1.7	Mobility bandgap (E_C-E_V)
N_{CV} (cm^{-3})	10^{19}	Effective density of states
N_T (cm^{-3})	$5.0\text{x}10^{18}$	Density of recombination centers
β (cm^3/s)	$1.62\text{x}10^{-9}$	Effective coupling coefficient
α_1, α_2 (1)	1	Symmetry parameters
E (kV/cm)	(V_{bi} - V_D)/d	Electric Field
kT (meV)	25.85	Thermal energy
V_{bi} (V)	0.8	Built-in voltage
V_D (V)	0.5	Applied bias voltage
d (nm)	100	Film thickness

Supplementary Table 1: Parameters used for the calculations in Supplementary Notes 1-3.

Method	Param	Fit		Unit	Description
Optical Absorption	E_g	= 1.37		[eV]	Optical band gap
Light IV	V_{oc}	= 0.51±0.1		[V]	Open-circuit voltage
	J_{sc}	= 14.7 ± 0.9		[mAcm^{-2}]	Short-circuit current
	FF	= 0.58 ± 0.01		[1]	Fill Factor
	η_{PCE}	= 4.31± 0.29		[%]	Power Conv. Efficiency
Dark IVT	n_{id}	= 1.62		[1]	Ideality factor
	E_μ	= 1.135 (= 0.83 E_g)		[eV]	Mobility band gap
	J_{00}	= 334		[mAcm^{-2}K^{-2}]	Red. saturation current
	E_{rs}	= 0.265	(0.606)	[eV]	Series res. act. energy.
	R^{-1}_{s00}	= 894	(1.3×10^9)	[Scm^{-2}]	Series res. prefactor
TAS	E_T	= 0.285		[eV]	Trap activation energy
	N_T	= 1.28×10^{17}		[cm^{-3}]	Trap state density
	E_{TF}	= 0.018		[eV]	Fermi to trap energy

Supplementary Table 2: Characterization summary for heterojunction device.

Method	Param.	Fit	Unit	Description
Optical Absorption	E_g	= 1.50	[eV]	Optical band gap
Light IV	V_{oc}	= 0.47±0.03	[V]	Open-circuit voltage
	J_{sc}	= 9.26 ± 0.77	[mAcm^{-2}]	Short-circuit current
	FF	= 0.32 ± 0.03	[1]	Fill Factor
	η_{PCE}	= 1.39± 0.32	[%]	Power Conv. Efficiency
Dark IVT	n_{id}	= 2.00	[1]	Ideality factor
	E_μ	= 1.133 (= 0.76 E_g)	[eV]	Mobility band gap
	J_{00}	= 0.78	[mAcm^{-2}K^{-2}]	Red. saturation current
	E_{rs}	= 0.38	[eV]	Series res. act. energy.
	R^{-1}_{s00}	= 168	[Scm^{-2}]	Series res. prefactor
TAS	E_T	= 0.27	[eV]	Trap activation energy
	N_T	= 1.69×10^{17}	[cm^{-3}]	Trap state density
	E_{TF}	= 0.028	[eV]	Fermi to trap energy

Supplementary Table 3: Characterization summary for BDT-device.

Symbol	Name
α, α_{PF}	Barrier-lowering constant
β	Effective capture coefficient
ε_s	Dielectric constant of NC-film
μ	Charge carrier mobility
σ_{PF}	Poole-Frankel conductivity
E_C	Conduction band energy
E_V	Valence band energy
E_g	(Optical) band gap
E_μ	Mobility band gap
E_{rs}	Series resistance activation energy
J_0	Saturation current
J_{00}	Reduced saturation current
J_{sc}	Short-circuit current
J_D	Device current
n_{id}	Ideality factor
N_C	Effective density of states (conduction band)
N_V	Effective density of states (valence band)
N_{CV}	Effective density of states (average)
N_T	Trap state density
R_p	Shunt resistance
R_s	Series resistance
R_{s0}	Series resistance (field independent)
R_{s00}	Series resistance prefactor
R^*	Capture radius of recombination center
V_D	Device voltage
V_{oc}	Open-circuit voltage
W_R	Width of recombination zone
Others	
e	Elementary charge
k	Boltzmann constant
T	Temperature

Supplementary Table 4: Overview over all parameters used in the main text and Supplementary Information.

Supplementary Note 1

We evaluated the goodness of fit of the current-voltage model (Eq. 1-4 in the main text) for different temperatures and voltage ranges by the coefficient of determination (R^2), which is defined as

$$R^2 = 1 - \frac{SS_{\text{res}}}{SS_{\text{tot}}}\,, \tag{7}$$

where the total data variance (SS_{tot}) and the residual sum of squared (SS_{res}) are given by

$$SS_{\text{res}} = \frac{1}{n}\sum_{i}^{n}\left(y_i - \hat{y}_i\right)^2 \text{ and } SS_{\text{tot}} = \frac{1}{n}\sum_{i}^{n}\left(y_i - \bar{y}\right)^2 \text{ and } \bar{y} = \frac{1}{n}\sum_{i}^{n} y_i \;. \tag{8}$$

The fitting was performed on the logarithm of the diode current, i.e. $y_i = \log(J_{\text{D},i})$.

In Supplementary Figure 7a, we show R^2 for the device shown in Figure 1 of the main text by taking into account only data points between 0 V and a maximum voltage V_{max}. For higher voltages, we find that the model starts to deviate more from the data (i.e. SS_{res} increases), but also the total data variance increases (SS_{tot}). The later effect dominates the R^2 value, so that R^2 improves for large voltage ranges up to 2V.

In Supplementary Figure 7b, we show R^2 for different temperatures down to 150K. While for 200K – 300K the R^2 value is 0.9946±0.0050, it starts to significantly decrease below 200K. We conclude that our model gives a very good description of the data above 200K.

Supplementary Note 2

In this section, we discuss the approximations used to simplify Eq. 5 in the main text:

$$J_{\mathrm{D}} = e\int_0^d U_{\mathrm{SRH}}(n, p, n_i, N_{\mathrm{T}}, E_{\mathrm{T}}, \beta_{\mathrm{n}}, \beta_{\mathrm{p}})dx$$

to Eq. 6 in the main text:

$$J_{\mathrm{D}} = \frac{e}{2} W_{\mathrm{R}}\, \beta N_{\mathrm{T}} N_{\mathrm{CV}} \exp\left[\frac{-E_{\mathrm{g}}}{2kT}\right]\left(\exp\left[\frac{-V_{\mathrm{D}}}{2kT}\right]-1\right)$$

Specifically, we assumed that (1) recombination occurs homogeneously in a region of width W_{R}, (2) the capture coefficients for electron and holes are equal ($\beta_{\mathrm{n}} = \beta_{\mathrm{p}} = \beta$), and (3) the effective density of states of the conduction and valence bands are equal ($N_{\mathrm{C}} = N_{\mathrm{V}} = N_{\mathrm{CV}}$).

To validate these assumptions, we (1) evaluate that the spatial profile of the recombination rate across the device thickness remains for different capture coefficients for electrons and holes (i.e. different values of $\beta = \sqrt{\beta_{\mathrm{n}}\beta_{\mathrm{p}}}$), different ratios between the electron and hole capture coefficients (i.e. different values of $\alpha_1 = \beta_{\mathrm{n}} / \beta_{\mathrm{p}}$), and different ratios between the conduction and valence band density of states (i.e, different values of $\alpha_2 = N_{\mathrm{C}} / N_{\mathrm{V}}$), and then (2) compare numerically evaluated values for recombination current over the parameter range of interest (i.e. Eq. 5 main text) to the analytical expression (i.e., Eq. 6 main text). Our discussion follows the main findings and notation in the work by Sah et al. [1] and Kirchartz et al.[2].

First we must write the recombination rate as a function of device thickness such that the spatial profile of recombination can be assessed and can be numerically integrated to evaluate the recombination current. Following the notation of Ref. 2, the recombination rate in the Shockley-Read-Hall-model is given by:

$$U_{\mathrm{SRH}} = \frac{\beta_n \beta_p N_{\mathrm{T}}(np - n_{\mathrm{i}}^2)}{\beta_n\left(n + N_{\mathrm{C}} \exp\left[\frac{E_{\mathrm{T}} - E_{\mathrm{C}}}{kT}\right]\right) + \beta_p\left(p + N_{\mathrm{V}} \exp\left[\frac{E_{\mathrm{V}} - E_T}{kT}\right]\right)}, \tag{1}$$

where the parameters are:

- The energy level of the conduction band (E_{C}), valence band (E_{V}), and the trap level (E_{T}),
- The effective density of states of the conduction band (N_{C}), valence band (N_{V}), and trap level (N_{T})

- The electron density (n), hole density (p), and intrinsic charge carrier density (n_i), and
- The capture coefficients for electrons (β_n) and holes (β_p)

We rewrite the above equation using the following substitutions

- $\alpha_1 = \beta_n / \beta_p$ (symmetry parameter of the capture coefficients)
- $\beta = \sqrt{\beta_n \beta_p}$ (geometric average of capture coefficient)
- $\alpha_2 = N_C / N_V$ (symmetry parameter of the effective density of states)
- $N_{CV} = \sqrt{N_C N_V}$ (geometric average of density of states)

and find:

$$U_{SRH} = \beta N_T N_{CV} \frac{\exp\left[\frac{E_\mu}{kT}\right](\exp\left[\frac{E_{Fn} - E_{Fp}}{kT}\right] - 1)}{(\alpha_1\alpha_2)^{1/2}(\exp\left[\frac{E_{Fn} - E_C}{kT}\right] + \exp\left[\frac{E_T - E_C}{kT}\right]) + (\alpha_1\alpha_2)^{-1/2}(\exp\left[\frac{E_V - E_{Fp}}{kT}\right] + \exp\left[\frac{E_V - E_T}{kT}\right])} \quad (2)$$

The total recombination current in the device is now given by the integral over the device thickness:

$$J_{RG} = e\int_{-d/2}^{+d/2} U_{SRH}(x)dx\,. \quad (3)$$

We solve this equation numerically using the following assumptions and parameters:

- α_1, α_2, β are independent of x
- Effective densities (N_{CV}, N_T) are independent of x
- The applied voltage is given by the difference of the quasi-Fermi levels:[1] $V_D = E_{Fn}$-E_{Fp}

For our calculations, we follow Ref. 1 and assume a constant electric field in the device and constant quasi-Fermi levels through the device (See Supplementary Figure 4a):

- $E_C(x) = -E\, x$
- $E_{Fn}(x) = E_C(0) - 0.5\,(E_\mu - V_D/2 + kT\,\mathrm{Log}(\alpha_2))$

If not otherwise mentioned, we use the parameter values shown in Supplementary Table 1.

The width of the recombination region

Sah et al.[1] find that the largest fraction of this recombination current occurs in a narrow region with the approximate width of:

$$W_{\mathrm{R,est}} = \pi kT /\, eE\,, \tag{4}$$

where E is the electric field. Assuming a homogeneous electric field over the film thickness in our devices, we estimate the electric field from the built-in voltage (V_{bi}): $E=(V_{\mathrm{bi}}-V_{\mathrm{D}})/d$.

We test this approximation by solving Supplementary Equation 3 numerically for different α_1, α_2, and β and plot $U_{\mathrm{SRH}}(x)$ in Supplementary Figure 4b-d. We see that the spatial width of the recombination rate W_{R} does not change for different ratios of electron and hole capture coefficients (α_1), different ratios of conduction and valence band density of states (α_2), and the value of the capture coefficient (β).

This can be understood from Supplementary Equation 2: In the denominator the factors $(\alpha_1\alpha_2)^{-1/2}$ and $(\alpha_1\alpha_2)^{+1/2}$ can be viewed equivalently as a shift of E_{C} and E_{V} by an energy $kT\log(\alpha_1\alpha_2)\,/\,2$. Therefore, in our calculation, the peak in the recombination shifts (but does not broaden) in Supplementary Figure 4c, d.

To arrive at the Eq. 6 in the main text, we followed Sah et al.[1] and approximate the integral in Supplementary Equation 3 as

$$J_{\mathrm{RG}} \approx J_{\mathrm{RG,est}} = \frac{e}{2} W_{\mathrm{R,est}} \times \beta N_{\mathrm{T}} N_{\mathrm{CV}} \exp\left[\frac{-E_{\mu}}{2kT}\right]\left(\exp\left[\frac{-V_{\mathrm{D}}}{2kT}\right]-1\right). \tag{5}$$

We assess the validity of this approximation in the relevant parameter range of this work by comparing the approximation to the numerically evaluated recombination current (Supplementary Equation 3) for 40 parameter sets selected randomly from the following distributions:

- E_{μ} uniform from [0.8,1.8] eV
- V_{bi} uniform from [0.5,1.0] V
- E_{T}-E_{i} uniform from [-0.2,+0.2] E_{g}
- log(α_1), log (α_2) uniform from [0.1,10]

for the relevant voltage and temperature range (V_D between 0.1 V and 0.4 V in steps of 0.05 V and T between 150 K to 300 K in steps of 30 K).

In Supplementary Figure 5a, we plot a histogram over all calculated values of $J_{RG}/J_{RG,est}$ and find that it is in most cases close to 1. We conclude that the approximation is good for the purposes of the discussion in the main text.

In our analysis of the size dependence of J_{00}, we assume that the effective density of states of the conduction and valence bands (N_C and N_V) scale very weakly with the NC size, due to the following argument: Based on Ref. 39, we expect two main effects to influence the effective density of states of the lowest energy mini-bands: (1) Larger NCs increase the volume of the unit-cell (b^3), such that the volume density of states reduces like $b^{-3} \sim r^{-3}$. (2) The width of the mini-band (Δ) broadens for smaller NCs due to the increased coupling of the wave functions. A broader band leads to a reduced effective density of states, due to the weighting by the Boltzmann-Factor. In Ref. 39, it is shown that Δ scales roughly like r^{-3}, so that both effects can be expected to approximately compensate for each other.

Parameter estimation

Finally, we assess how well the charge transport parameters J_{00}, E_μ, and n_{id} can be estimated by fitting Eq. 1-2 (Ref. 3) to measurement data. We use the numerically calculated J_{RG} with randomly chosen device parameters (E_g, E_{bi}, E_T, α_1, α_2) and fit Eq. 1-2 from the main text (for $R_s = R^{-1}_p = 0$) to the J_{RG} data by adjusting the parameters J_{00}, E_μ, n_{id}.
Since J_{00} does not enter the simulation directly we calculate the theoretical value for J_{00} from the simulation parameters. Using the equations developed in the main text, $J_{00}T^2 = \frac{e}{2} W_R \beta N_T N_{CV}$,

$\beta = 4\pi R^* \frac{kT}{e}\mu$, and $W_{R,est} = \pi kT / qE = d \times \pi kT / (eV_{bi} - eV_D)$, we find

$$J_{00} = \left(2\pi^2 k^2\right) \frac{N_T N_{CV}}{qE} R^* \mu \quad [\text{A cm}^{-2}\ \text{K}^{-2}], \tag{6}$$

where k is the Boltzmann constant and the temperature dependence of T^2 cancels.

In Supplementary Figure 6a, we compare the J_{00} that was estimated by fitting from the simulated data with the theoretical value based on the input parameters for the simulation (Supplementary Equation 6) and find good agreement. In other words, the parameter J_{00} can be estimated well from the J_{RG} data for a broad range of parameters. The spread along the unity line (gray) results from the random variation of the built-in voltage.

In Supplementary Figure 6b, we show the ratio of the E_μ estimated by fitting from the simulated data and the E_μ used in the simulation of the J_{RG} data, as a function of the ideality factor (n_{id}). For ideality factors close to 2, we find a ratio of ~1 indicating that E_μ is estimated well from the simulated data. For lower ideality factors ($n_{id} < 2$) the mobility band gap (E_μ) will be underestimated from its real value, but our analysis shows that the error in the estimation of E_μ is in all simulated cases limited to less than 25% of the correct value of E_μ.

Supplementary Note 3

The trend of the free carrier mobility with NC size, showing a strong increase in mobility with decreasing NC size, that is predicted theoretically and found to be in quantitative agreement with our measurements of J_{00} and R^{-1}_{s00}, may at first seem contradictory to previous experimental work, which reported a decreasing or non-monotonic trend with increasing band gap (decreasing NC size).[5–7] Here we explain the reasons behind this seeming inconsistency.

In most studies, carrier mobility is measured in field-effect transistors (FET) structures, where mobility is determined by dividing the source-drain-current by the total charge carrier density (often written as the oxide-capacitance) and accounting for the geometry. Since there is no differentiation of the fractions of charge carriers that are free and trapped, the obtained mobility is an effective mobility.[8] The fraction of free versus trapped charge carriers is exponentially dependent on the energy difference between the trap-states and the conduction band. Because this energy difference increases with larger NC band gap (as seen from our measurements of E_{rs} and E_T), a significantly smaller fraction of charge carriers are free in films composed of larger band gap NCs. In this situation, the effective mobility is measured to decrease even if the free charge carrier mobility increases for larger band gap NC-solids. Said differently, the increase in mobility is compensated by a larger activation energy. This leads to non-monotonic trends in the effective mobility as reported by Liu *et al.*[7] and is analogous to our measurement of the series resistance R_{s0}, which includes both R_{s00} and E_{rs}, shown in Fig. 4b.

It should further be noted that in other regimes of charge transport, different effects are important. In particular, the semi-metallic regime with charge carrier densities of 1 electron per NC (~10^{19} cm^{-3}), which is about 2-3 orders of magnitude higher than in our solar cell devices, it was shown that charge carrier mobility is not anymore limited by NC-NC coupling, but by the charging energy of a NC.[5,6]

Supplementary Note 4

We demonstrate the generality of our findings in the main text by applying our analysis methods and model to a heterojunction device with 1,2-ethanedithiol as a cross-linking ligand and a MSM device using 1,4-benzenedithiol as a cross-linking ligand.

The heterojunction device has the following structure:

ITO / TiO_2 (100nm) / PbS:EDT (165nm) / MoOx (10nm) / Au (30nm) / Ag (500nm)

Following Ref. 4, a titania nanoparticle paste (DSL 90-T, Dyesol), diluted to 125 mg/mL in acetone, was spun at 2500 rpm for 60 s on cleaned ITO substrates. The titania films were annealed in air at 500ºC for 60 min. PbS NC-layers were deposited by the same sequential dip-coating procedure used for the MSM devices. 60 dip coating cycles were performed, resulting in device thicknesses of 165±3nm, as measured from SEM cross-sections. MoOx (10nm)/Au (30nm)/Ag (500nm) top electrodes were deposited by thermal evaporation.

In Supplementary Figure 8, we show the basic characterization of the NCs and the current-voltage characteristics of the device in dark and under AM1.5G illumination. Supplementary Figure 9 shows temperature dependent current-voltage (IVT) and thermal admittance spectroscopy (TAS) data. All charge transport parameters extracted from these measurements are summarized in Supplementary Table 2.

In agreement with our findings for the ethanedithiol MSM diode in the main text, we find that trap-assisted recombination can explain the diode current (n_{id} = 1.62) and that the mobility band gap is close to the optical band gap (E_μ = 0.83 E_g). Further, the series resistance follows the barrier lowering shape of Eq. 3 in the main text. Both the activation energy (E_{rs} = 0.26 eV) and prefactor ($R^{-1}{}_{s00}$ = 894) are in agreement with the data in Figure 2 in the main text. The trap energy (E_{T1} = 0.285eV) determined by TAS matches the series resistance (E_{rs} = 0.26 eV).

In contrast to the MSM devices, we observe a second activation energy of the series resistance at lower temperatures ($E_{rs,2}$ = ~0.6 eV). The occurrence of two activation energies can arise from the fact that the Fermi-level is pinned only in one portion of the device, which is supported by the finding that the effective distance (d) of the Poole-Frankel conduction region is shorter (60nm)

than the total film (160nm). The value for J_{00} = 334 is also high compared to the values obtained for the MSM diode. We attribute this in part to a lower electric field, which leads to a wider recombination zone (See Supplementary Equation 6). In summary, the data from the heterojunction device can be satisfactorily explained with the model developed for MSM diodes.

For the device using the 1,4-Benzenedithiol ligand (BDT), we use the same procedure described in the main text to fabricate a MSM-device. The crosslinking solution in this case consists of 10mM BDT in anhydrous acetonitrile.

In Supplementary Figure 10, we show the basic characterization of the NCs and the current-voltage characteristics of the device under AM1.5G illumination. In Supplementary Figure 11 we show temperature dependent current-voltage (IVT) and thermal admittance spectroscopy (TAS) data. All charge transport parameters extracted from these measurements are summarized in Supplementary Table 3.

In agreement with the main text we find that trap-assisted recombination can explain the diode current (n_{id} = 2.00) and that the mobility band gap is close to the optical band gap (E_{μ} = 0.76 E_g). The prefactor J_{00} = 0.78, is in good agreement with the data in Figure 1f. Further, we find the series resistance to follow the barrier lowering shape of Eq. 3 in the main text. Both activation energy (E_{rs} = 0.38 eV) and prefactor (R^{-1}_{s00} = 168) are in agreement with the data in Figure 2 in the main text.

In contrast to the MSM devices, we find that the trap energy determined by TAS (E_{T1} = 0.27eV) is slightly smaller than the activation energy of the series resistance (E_{rs} = 0.38 eV). Also the ratio of the mobility band gap to the optical band gap is found to be slightly lower than for the EDT devices (E_{μ}/E_g = 0.76).

The data from the BDT-device is in line with the main points developed in the main text.

Supplementary Note 5

For the convenience of the reader, we summarize all equations used for data fitting and interpretation of temperature-dependent current-voltage data. An overview of all parameters is given in Supplementary Table 4.

Data Fitting:

$$J_{\mathrm{D}} = \frac{R_{\mathrm{p}}}{R_{\mathrm{s}} + R_{\mathrm{p}}} \left\{ J_0 \left[\exp\left(\frac{e(V_{\mathrm{D}} - J_{\mathrm{D}} R_{\mathrm{s}})}{n_{\mathrm{id}} kT} \right) - 1 \right] \right\} + \frac{V_{\mathrm{D}}}{R_{\mathrm{p}}} + J_{\mathrm{sc}} \qquad J_0 = J_{00} T^2 \exp\left[-\frac{E_\mu}{n_{\mathrm{id}} kT} \right]$$

$$R_{\mathrm{s}} = R_{\mathrm{s0}} \exp\left[\frac{\alpha \sqrt{V_{\mathrm{D}} / d}}{kT} \right] \qquad R_{\mathrm{s0}} = R_{\mathrm{s00}} \exp\left[\frac{E_{\mathrm{rs}}}{kT} \right]$$

Physical Models:

Shockley-Read-Hall recombination current:

$$J_{\mathrm{D}} = \frac{e}{2} W_{\mathrm{R}}\, \beta N_{\mathrm{T}} N_{\mathrm{CV}} \exp\left[\frac{-E_{\mathrm{g}}}{2kT} \right] \left(\exp\left[\frac{-V_{\mathrm{D}}}{2kT} \right] - 1 \right)$$

Relation to data fitting equations:

$n_{\mathrm{id}} = 2$, $E_\mu = E_{\mathrm{g}}$, and $J_{00} T^2 = \frac{e}{2} W_{\mathrm{R}}\, \beta N_{\mathrm{T}} N_{\mathrm{CV}}$

Diffusion limited capture coefficient:

$$\beta = 4\pi R^* \frac{kT}{e} \mu$$

Poole-Frenkel:

$$\sigma_{\mathrm{PF}}(F, T) = e\mu N_{\mathrm{C}} \exp\left[\frac{E_{\mathrm{C}} - E_F}{kT} \right] \exp\left[\frac{\alpha_{\mathrm{PF}} \sqrt{F}}{kT} \right]$$

$$\alpha_{\mathrm{PF}} = \sqrt{e^3 / (\pi \varepsilon_{\mathrm{s}})}$$

Relation to data fitting equations (using: $R^{-1}{}_{\mathrm{s}} = \sigma_{\mathrm{PF}}/d$ and $F = V_{\mathrm{D}}/d$)

$$R_{\mathrm{s00}}^{-1} = e\mu N_{\mathrm{C}} / d$$

Supplementary References

1. Sah, C., Noyce, R. & Shockley, W. Carrier generation and recombination in P-N junctions and P-N junction characteristics. *Proc. IRE* **45,** 1228–1243 (1957).

2. Kirchartz, T., Pieters, B. E., Kirkpatrick, J., Rau, U. & Nelson, J. Recombination via tail states in polythiophene:fullerene solar cells. *Phys. Rev. B* **83,** 115209 (2011).

3. Rau, U. & Schock, H. Electronic properties of Cu(In,Ga)Se_2 heterojunction solar cells-recent achievements, current understanding, and future challenges. *Appl. Phys. A Mater. Sci. Process.* **69,** 131–147 (1999).

4. Pattantyus-Abraham, A. G. *et al.* Depleted-heterojunction colloidal quantum dot solar cells. *ACS Nano* **4,** 3374–80 (2010).

5. Kang, M. S., Sahu, A., Norris, D. J. & Frisbie, C. D. Size-dependent electrical transport in CdSe nanocrystal thin films. *Nano Lett.* **10,** 3727–32 (2010).

6. Kang, M. S., Sahu, A., Norris, D. J. & Frisbie, C. D. Size- and temperature-dependent charge transport in PbSe nanocrystal thin films. *Nano Lett.* **11,** 3887–92 (2011).

7. Liu, Y. *et al.* Dependence of carrier mobility on nanocrystal size and ligand length in PbSe nanocrystal solids. *Nano Lett.* **10,** 1960–9 (2010).

8. Stallinga, P. *Electrical Characterization of Organic Electronic Materials and Devices*. 8–9 (John Wiley & Sons, Ltd, 2009).
